# Optimization of temporal sampling for ^18^F-choline uptake quantification in prostate cancer assessment

**DOI:** 10.1186/s13550-018-0410-8

**Published:** 2018-06-15

**Authors:** Xavier Palard-Novello, Anne-Lise Blin, Florence Le Jeune, Etienne Garin, Pierre-Yves Salaün, Anne Devillers, Giulio Gambarota, Solène Querellou, Patrick Bourguet, Hervé Saint-Jalmes

**Affiliations:** 1 LTSI-UMR1099, Univ Rennes, Inserm, F-35000 Rennes, France; 2 Department of Nuclear Medicine, Centre Eugène Marquis, Rennes, France; 3 Univ Rennes-EA 4712, Rennes, France; 4 UMR 124, Univ Rennes, Inserm, Rennes, France; 5 Department of Nuclear Medicine, Centre Hospitalier Universitaire, Brest, France; 6 University of Bretagne Occidentale-EA 3878, Brest, France

**Keywords:** ^18^FCholine, Positron emission tomography, Prostate cancer, Kinetic analysis

## Abstract

**Background:**

Suboptimal temporal sampling of time-activity curves (TAC) from dynamic ^18^F-fluoromethylcholine (FCH) PET images may introduce bias in quantification of FCH uptake in prostate cancer assessment. We sought to define an optimal temporal sampling protocol for dynamic FCH PET imaging.

Seven different time samplings were tested: 5 × 60″, 10 × 30″, 15 × 15″–1 × 75″, 6 × 10″–8 × 30″, 12 × 5″–8 × 30″; 10 × 5″–4 × 10″–3 × 20″–5 × 30″, and 8 × 3″–8 × 12″–6 × 30″. First, the irreversible and reversible one-tissue compartment model with blood volume parameter (VB) (respectively, 1T1K+VB and 1T2k+VB, with K1 = transfer coefficient from the arterial blood to the tissue compartment and k2 = transfer coefficient from the tissue compartment to the arterial blood) were compared for 37 lesions from 32 patients who underwent FCH PET imaging for initial or recurrence assessment of prostate cancer, and the model was selected using the Akaike information criterion. To determine the optimal time sampling, K1 values extracted from 1000 noisy-simulated TAC using Monte Carlo method from the seven different time samplings were compared to a target K1 value which is the average of the K1 values extracted from the 37 lesions using an imaging-derived input function for each patient. K1 values extracted with the optimal time sampling for each tumoral lesion were compared to K1 values extracted from each of the other time samplings for the 37 lesions.

**Results:**

The 1T2k + VB model was selected. The target K1 value as the objective was 0.506 mL/ccm/min (range 0.216–1.246). Results showed a significant difference between K1 values from the simulated TAC with the seven different time samplings analyzed. The closest K1 value from the simulated TAC to the target K1 value was obtained by the 12 × 5″–8 × 30″ time sampling. Concerning the clinical validation, K1 values extracted from the optimal time sampling (12 × 5″–8 × 30″) were significantly different with K1 values extracted from the other time samplings, except for the comparison with K1 values extracted from the 10 × 5″–4 × 10″–3 × 20″–5 × 30″ time sampling.

**Conclusions:**

A two-phase framing of dynamic PET reconstruction with frame durations of 5 s (blood phase) and 30 s (tissue phase) could be used to sample the TAC for uptake quantification in prostate cancer assessment.

## Background

Prostate cancer (PC) is the most commonly diagnosed cancer in males worldwide [1]. Many results showed the usefulness of ^18^F-labeled choline (FCH) tracers for non-invasive positron emission tomography/computed tomography (PET/CT) in PC [2, 3]. Choline is a precursor of the biosynthesis of phosphatidylcholine, which is located on the cell membrane phospholipids and highly expressed in cancer, especially in PC [4–6]. Usually, the imaging protocol for FCH PET consists of a dual-phase procedure: a pelvic acquisition starts immediately after tracer injection followed by a late scan covering the base of the skull through the superior portion of the thighs [7–9]. The early phase is mainly used in order to detect pelvic lesions before the radioactive urine appears in the excretory pathways [7, 10, 11]. Moreover, kinetic parameters extracted from the early acquisition could add further information concerning tumor aggressiveness [12, 13]. Information concerning tumor aggressiveness from a non-invasive imaging procedure could be used to guide biopsy [14] and potentially improve patient management with dose escalation using intensity-modulated radiotherapy [15]. PET imaging using list-mode acquisition is now performed on modern PET systems. However, this massive list-mode data collection cannot be stored on a clinical picture archiving and communication system (around 1.3 GB for an acquisition of 10 min). So, a time sampling is needed before reconstruction of the list-mode data for the kinetic analysis. Concerning this time sampling for the kinetic analysis, no recommendations are available at the moment. Authors who published results with FCH kinetic analysis in PC were using different time samplings [16–19], with time bins ranging from 5 s to several minutes or more. The quantification of FCH uptake with kinetic analysis mandates careful optimization of time sampling. While shorter time bins will increase temporal resolution, which may be of interest during the first minutes for a better definition of the early blood peak, shorter time bins also result in decreased image quality due to lower total counts per image and increased reconstruction time and storage requirements for reconstructed images. On the contrary, insufficient temporal resolution results in under-sampling of arterial input function and may produce biased estimates of FCH influx. We sought to define an optimal temporal sampling protocol for dynamic FCH PET imaging in modern PET systems using real-world data from patients and simulations.

## Methods

### Time sampling

Seven different time samplings with a total study duration of 5 min were compared. Three time samplings were based on previous studies: 10 × 30″ [17], 6 × 10″–8 × 30″ [19], and 10 × 5″–4 × 10″–3 × 20″–5 × 30″ [16]. In addition, four time samplings with different frame durations were arbitrarily chosen: 5 × 60″, 15 × 15″–1 × 75″, 12 × 5″–8 × 30″, and 8 × 3″–8 × 12″–6 × 30″.

### Clinical analysis

#### Patients

Patients with histologically proven PC from September 2016 to May 2017 referred to our department for initial or recurrence PC assessment were included. Exclusion criteria were a lesion out of the early pelvic field-of-view (FOV), a size lesion < 0.7 cm^3^ (to avoid partial volume effect), and FCH uptake in the inguinal region (interpreted as inflammatory benign lesion as previously discussed in the literature [20–22]).

#### PET/CT imaging protocol

**Fig. 1:**
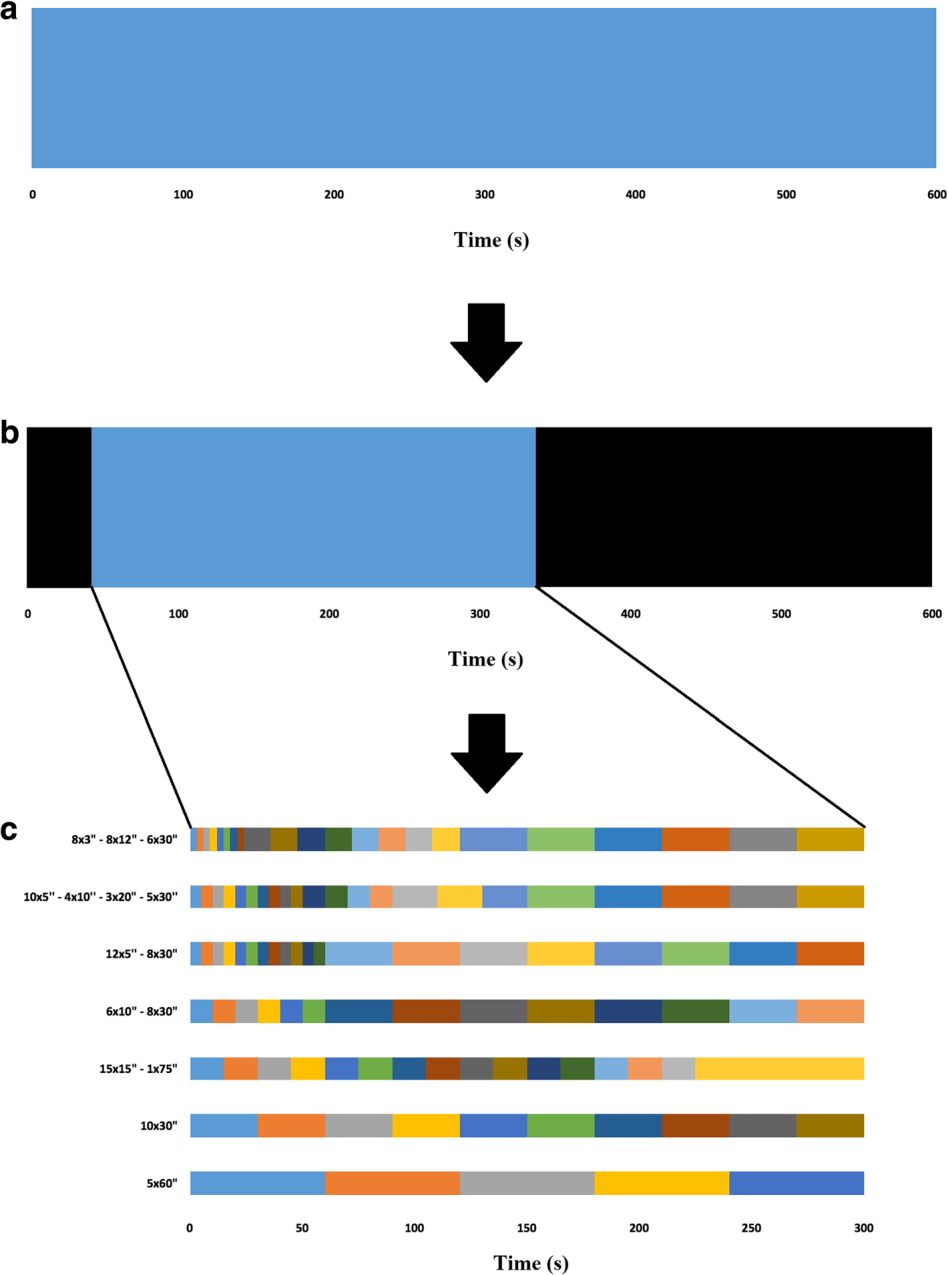
Among the 600 s of the PET acquisition (**a**), the first seconds without any count were excluded and only 5 min from the arrival time of the FCH bolus were selected for each patient (**b**). Then, PET data were reconstructed into the seven different time samplings for each patient (**c**)

Each patient underwent a CT scan followed by a 10-min PET scan using list-mode acquisition with the FOV centered over the pelvic region (Siemens Biograph mCT, Knoxville, TN, USA). At the start of the PET scan, 3 MBq/kg [23–25] of FCH was administered intravenously. All patients fasted at least 6 h before the FCH PET/CT scan [23, 24]. A whole-body PET/CT scan was performed 1 h post injection. PET data were reconstructed using point spread function-based time of flight 3D ordered-subset expectation maximization iterative algorithm (2 iterations, 21 subsets) with corrections (attenuation, dead time, randoms, scatter, and decay) and a 2-mm kernel convolution filter. Voxel size was 4 × 4 × 2 mm^3^. First, a 10-min static image was reconstructed. Second, PET data were reconstructed into 20 frames of 3 s (lower bound of time bin reconstruction available on the system) in order to determine the arrival time of the FCH bolus for each patient. Then, PET data were reconstructed into the seven different time samplings during 5 min from the bolus arrival time (Fig. 1).

#### Image analysis

Tumoral and arterial time-activity curves (TAC) were generated by a nuclear medicine physician with the Syngo.via software (Siemens). A freehand tumoral volume of interest (VOI) with a threshold of 40% of the maximum signal intensity was drawn on the 5-min static reconstruction and projected onto each frame of the seven different time samplings. An imaging-derived input function (IDIF) was estimated from a manually drawn VOI within the external iliac artery on the early PET image in which the peak blood pool activity was the highest, helped with CT scan. The standardized uptake value (SUV) was calculated and adjusted by means of an injected dose according to tissue activity concentration and patient body weight. The SUVmean of the tumor VOI was measured on the 5-min static reconstruction.

#### Kinetic model selection

To extract kinetic parameters (PMOD software version 3.8; PMOD Technologies; Zürich, Switzerland), the one-tissue compartment model (with K1 = transfer coefficient from the arterial blood to the tissue compartment and k2 = transfer coefficient from the tissue compartment to the arterial blood) with a blood volume parameter (VB) reversible (1T2k + VB) and irreversible (1T1k + VB) were applied [16, 19]. The model providing the best fits (Levenberg-Marquadt algorithm) to the tumoral TAC with the seven different time samplings was selected on the basis of the Akaike information criterion (AIC) for small sample sizes [26].

### Optimal time sampling

To investigate the optimal time sampling between the seven proposed time samplings, Monte Carlo simulations were performed in Mathematica (Wolfram Research, Inc., Mathematica, Version 11.1, Champaign, IL, USA (2017)). A modeled arterial TAC (C_IDIF_(t)) was obtained from the mean of the arterial TAC from the fastest initial temporal sampling (8 × 3″–8 × 12″–6 × 30″) extracted from the patients with interpolation to 1-s frames. This modeled arterial TAC was applied for every investigated time sampling. Average K1 and average k2 parameters extracted from the lesions with the seven different time samplings according to the selected model provided a modeled tumoral TAC C(t) as follows:

$$ \mathrm{C}\left(\mathrm{t}\right)=\mathrm{VB}\ {\mathrm{C}}_{\mathrm{IDIF}}\left(\mathrm{t}\right)+\left(1-\mathrm{VB}\right)\ \mathrm{K}1\ {\mathrm{e}}^{-\mathrm{k}2\mathrm{t}}\otimes {\mathrm{C}}_{\mathrm{IDIF}}\left(\mathrm{t}\right) $$

For each time sampling, 1000 realizations of independent distributed Poisson noise (added noise) were added to the modeled TAC as follows:

$$ \mathrm{Added}\ \mathrm{noise}=c\ \left(\mathrm{RandomInteger}\left[\mathrm{PoissonDistribution}\left[\mathrm{C}\left(\mathrm{t}\right)\right]\right]-\mathrm{C}\left(\mathrm{t}\right)\right)/\mathrm{Sqrt}\left(\mathrm{dt}\right), $$

where *c* is the scaling factor and dt is the frame duration.

Each realization was fitted to the model providing an estimation of the kinetic parameters. The mean and standard deviation of the estimated K1 values were computed from all the realizations and compared to the target K1 value. The target K1 value was the average of K1 values extracted for all of the lesions from all of the time samplings with the selected model.

### Clinical validation

To confirm the difference between K1 values from each time sampling, we compared K1 values for each of the 37 lesions extracted from the optimal time sampling with K1 values extracted from the other time samplings using the Wilcoxon test for paired samples (Wolfram Research, Inc., Mathematica, Version 11.1, Champaign, IL, USA (2017)). Two-sided values of *p* < 0.05 were considered significant.

### Optimal time sampling for the tissue phase

Concerning the tissue phase, we applied the same methods as described previously (“Optimal time sampling” section). Five different time samplings based on the best optimal time sampling found previously with changes of time binning only for the late phase following the arterial peak activity were applied.

### Correlation between K1 and static parameter

K1 values extracted from the optimal time sampling for each of the lesions were correlated with SUVmean (10-min static reconstruction) using a Spearman rank correlation test.

## Results

### Clinical analysis

#### Patients

**Fig. 2:**
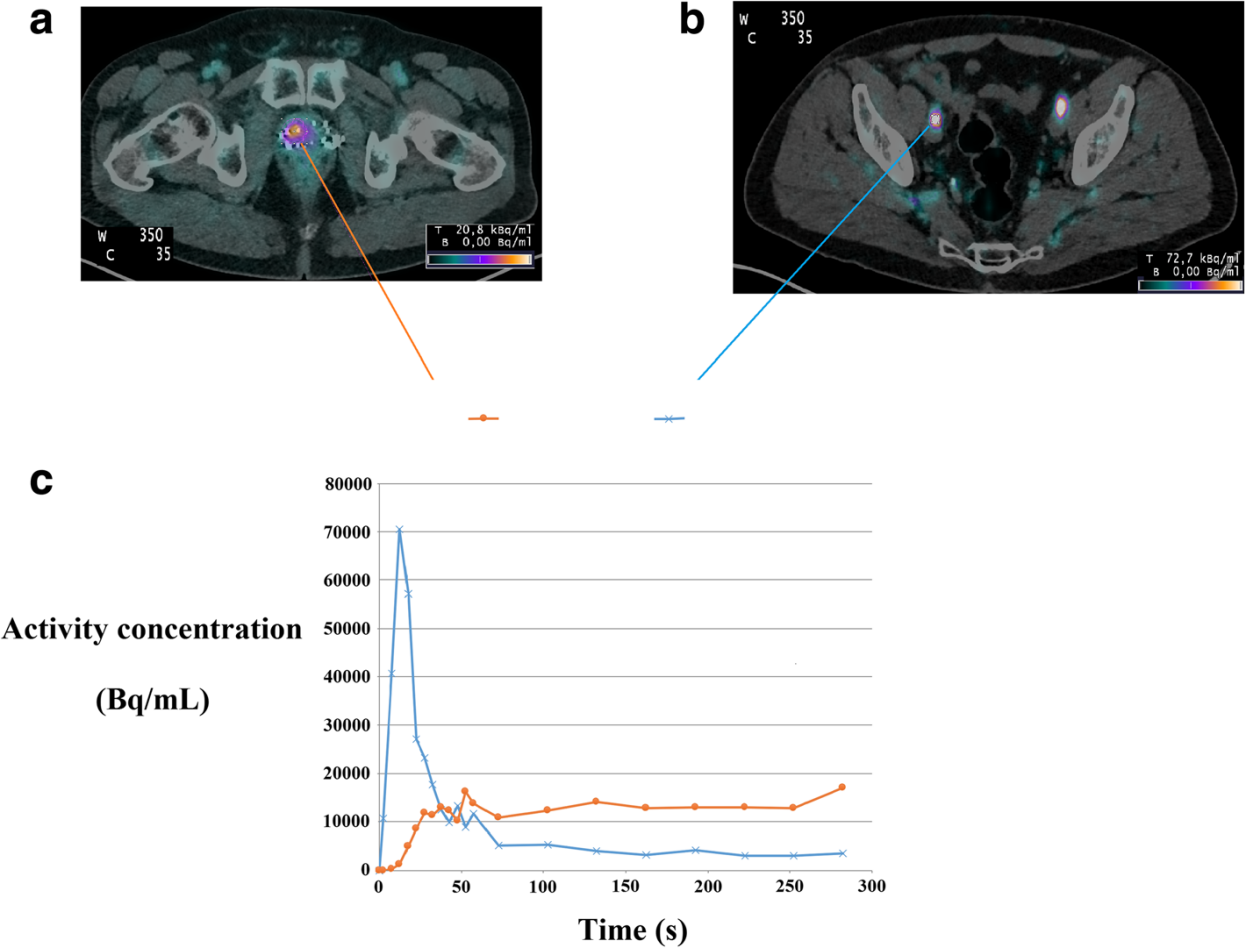
Fused axial FCH PET/CT images demonstrates prostatic lesion uptake in a 69-year-old man with PSA level = 5.5 ng/mL (**a**) and FCH bolus on the external iliac arteries (**b**) with corresponding arterial and lesion time-activity curves (**c**)

Ninety patients were included. Thirty-four patients had at least one lesion > 0.7 cm^3^ in the FOV of the early pelvic acquisition. Dynamic acquisition was unsuccessful for two patients. Therefore, 32 patients with 37 lesions were finally analyzed: 24 intra-prostatic lesions, 5 lesions of the prostatic bed, 1 lymph node lesion, and 7 bone lesions. Six patients were referred for initial assessment and 26 patients for recurrence of PC. Median age was 72 years (range 55–84). The Gleason score of the patients were as follows: 6 patients as Gleason 6, 13 patients as Gleason 7, 5 patients as Gleason 8, and 5 patients as Gleason 9. The Gleason score was not retrieved retrospectively for three patients who performed FCH PET/CT for prostate cancer recurrence initially diagnosed before 2010. At the time of FCH PET/CT, the median PSA level was 6.7 ng/mL (range 0.2–55.0). The median volume of the lesions was 4.2 cm^3^ (range 0.77–23.4). The median SUVmean of the lesions was 3.6 (range 2–8.9). Typical TAC in a 69-year-old man are shown in Fig. 2. So, we performed 224 reconstructions using list-mode acquisitions (32 patients and 7 different time samplings).

#### Kinetic model selection

AIC results indicated that the 1T2k+VB model produced the best fits (preferred model in 208 (80%) of the 259 lesions TAC from all of the time samplings). The average K1 value according to the 1T2k+VB model for all of the lesions from all of the time samplings was 0.506 mL/ccm/min ± 0.176 (range 0.216–1.246). The average k2 was 0.150 min^− 1^ ± 0.08 (range 0.008–0.402).

### Optimal time sampling

The parameters used for the modeled tumoral TAC were K1 = 0.506 min^−1^ and k2 = 0.150 min^−1^ using the 1T2K+VB model. Results showed a significant difference between the K1 values extracted from the simulated TAC with the seven different time samplings. The closest average K1 of the 1000 simulations to the target K1 value as objective (0.506 mL/ccm/min) is obtained by the 12 × 5″–8 × 30″ time sampling (Table 1).

**Table 1:** Comparison of the K1 values from the 1000 simulations (Monte Carlo) using the same IDIF for the seven different time samplings

Time sampling	Average K1 value (mL/ccm/min^−^) ± SD	95% confidence interval (mL/ccm/min)	
		Lower bound	Upper bound
5 × 60″	0.6474 ± 0.0188	0.6462	0.6486
10 × 30″	0.6126 ± 0.0176	0.6115	0.6137
15 × 15″–1 × 75″	0.5498 ± 0.0172	0.5487	0.5509
6 × 10″–8 × 30″	0.5191 ± 0.0148	0.5181	0.5200
12 × 5″–8 × 30″	0.5053 ± 0.0140	0.5044	0.5062
10 × 5″–4 × 10″–3 × 20″–5 × 30″	0.5073 ± 0.0140	0.5064	0.5082
8 × 3″–8 × 12″–6 × 30″	0.4982 ± 0.0137	0.4973	0.4990

### Clinical validation

For the 37 lesions, comparisons of K1 values from the optimal time sampling (12 × 5″–8 × 30″) with the rest of the time samplings tested showed significant differences for all of comparisons, except for the comparison with the 10 × 5″–4 × 10″–3 × 20″–5 × 30″ time sampling (Table 2).

**Table 2:** Comparison of the K1 values from the optimal time sampling (12 × 5″-8 × 30″) and K1 values from the other time samplings for clinical validation

Time sampling	Average K1 value (mL/ccm/min) ± SD	*p* value
5 × 60″	0.600 ± 0.232	< 0.001
10 × 30″	0.573 ± 0.184	< 0.001
15 × 15″–1 × 75″	0.511 ± 0.167	< 0.001
6 × 10″–8 × 30″	0.480 ± 0.150	< 0.001
12 × 5″–8 × 30″	0.465 ± 0.149	N/A
10 × 5″–4 × 10–3 × 20″–5 × 30″	0.463 ± 0.145	0.644
8 × 3″–8 × 12″–6 × 30″	0.453 ± 0.139	< 0.001

N/A not applicable

### Optimal time sampling for the tissue phase

In addition to the optimal time sampling (12 × 5″–8 × 30″), four time samplings with different frame durations for the tissue phase after the peak activity were arbitrarily chosen: 12 × 5″–16 × 15″, 12 × 5″–4 × 60″, 12 × 5″–2 × 120″, and 12 × 5″–1 × 240″. Results showed a significant difference between the K1 values extracted from the simulated TAC with the five different time samplings (Table 3). The closest average K1 of the 1000 simulations to the clinical K1 value as the objective (0.506 mL/ccm/min) is obtained by the 12 × 5”–8 × 30” time sampling.

**Table 3:** Comparison of the results of K1 values from the 1000 simulations (Monte Carlo) for the five different time sampling simulated for the tissue phase analysis

Time sampling	Average K1 value (mL/ccm/min) ± SD	95% confidence interval (mL/ccm/min)	
		Lower bound	Upper bound
12 × 5″–16 × 15″	0.5075 ± 0.0137	0.5067	0.5084
12 × 5″–8 × 30″	0.5053 ± 0.0140	0.5044	0.5062
12 × 5″–4 × 60″	0.4994 ± 0.0165	0.4984	0.5005
12 × 5″–2 × 120″	0.4988 ± 0.0235	0.4973	0.5002
12 × 5″–1 × 240″	0.5235 ± 0.0341	0.5213	0.5256

### Correlation between K1 and static parameter

Results showed that K1 values from the 12 × 5″–8 × 30″ time sampling were moderately correlated with SUVmean (*r* = 0.60; *p* < 0.001).

## Discussion

This study optimizes dynamic time frame binning during image reconstruction for quantification of FCH influx in prostate cancer assessment. Comparing protocols with different frame durations, results show that the 12 × 5″–8 × 30″ time framing is optimal among the seven different time framing analyzed.

Two studies recently evaluated a lesion-based correlation of quantified FCH uptake with tumor grade using full quantification. On the one hand, Schaefferkoetter et al. demonstrated that FCH influx was significantly higher in tumors with GS of 4+3 than that in tumors with GS of 3+4 or 3+5 [12]. On the other hand, Choi et al. found no significant associations of K1 influx with pathologic characteristics [19]. A different dynamic time binning protocol between these two previous studies might be a possible explanation. Under-sampling or over-sampling of time framing may produce biased estimates of PET full quantification [27]. That is the reason why optimal time frame binning was assessed concerning other PET tracers [27–29].

Concerning FCH, to the best of our knowledge, this is the first report assessing optimal time frame binning. To date, no guidelines are available and studies published concerning FCH were using different time samplings [16–19]. Nevertheless, finding an optimal time sampling is important due to the size of the list-mode data (for example, the 12 × 5″–8 × 30″ time binning data size is 230 MB vs 1.3 GB for the list-mode data).

Firstly, our results suggest that the better estimation of FCH quantification is obtained using an initial time frame of 5 s. Initial time frame longer than 5 s is not optimal for quantification, due to the increasing of the full width at half maximum (FWHM) of the blood pool TAC if time frames are lengthened. This under-sampling of the arterial input function produces biased estimates of quantification. However, results show that the increasing of temporal resolution with the fastest initial time frame (3 s) is not optimal neither to better estimate quantification. The low count rate during shorter time bins is probably the main reason of biased estimates of quantification, even when a modern PET system was used with better count statistics than older PET systems. These results are expected because the signal-to-noise ratio in PET data is roughly proportional to the square root of the number of counts (Poisson-distributed data). That is the reason why our results showed that a compromise has to be found between longer frames with better counting statistics but poor temporal resolution and shorter frames with poor counting statistics but better temporal resolution, which are consistent with the results suggested in studies with other PET tracers [27–29].

Secondly, concerning the tissue phase after the initial blood phase, our results demonstrated that a frame duration of 30 s seems to be optimal. The optimal sampling for the tissue phase is slower than the optimal sampling for the blood phase, which could be explained by the low variations in the tracer uptake during this phase and also by the lower count rate detected during the tissue phase than the count rate detected during the blood phase, so a longer time frame is needed in this phase. However, further studies are needed to confirm these results because no clinical validation was performed for this tissue phase in our study.

Thirdly, the results of the clinical validation confirm that a full quantification mandates careful optimization of time sampling. For the 37 lesions, when comparing K1 values from the optimal time sampling (12 × 5″–8 × 30″) with K1 values from the other time samplings for each of the 37 lesions, results show that FCH quantification was significantly different, except when comparing K1 values from the optimal time sampling (12 × 5″–8 × 30″) with K1 values from the 10 × 5″–4 × 10″–3 × 20″–5 × 30″ time sampling. The similar K1 results from these both time samplings with the same initial time binning suggest that optimizing the initial part is the most important.

Fourthly, in the current study, we observed a moderate correlation between K1 values extracted from the optimal time sampling (12 × 5″–8 × 30″) for each of the 37 lesions and SUVmean (*r* = 0.6). This result is consistent with those of previous studies [13, 16, 17, 19]. However, these results are not consistent with those of two previous studies showing high correlation between K1 and SUV [18, 30]. Two-tissue compartment models were used in these both latter studies and could be a potential reason for discrepancy with our results using a one-tissue compartment model.

This study has several limitations. First, an imaging-derived arterial input function was used for the kinetic modeling instead of a conventional plasma-derived input function, so accurate measurements of the radiolabeled metabolites were not possible. However, the metabolite fraction is very low during the first minutes after injection [31].

In traditional kinetic modeling, a plasma-derived input function is usually obtained from arterial sampling with a metabolite correction, which is relatively invasive and complex to perform in a routine clinical setting. However, Verwer et al. recently reported that the use of an imaging-derived plasma input function was feasible for a kinetic analysis [16], so it was used in other studies [12, 19]. Second, although the noise in projection data is Poisson distributed, the distribution is usually much more complex after the reconstruction process [32]. Third, histological confirmation of the prostatic lesions was obtained in 43% of lesions. Fourthly, K1 values were obtained using a current-generation 3D scanner with high count rate capabilities. For count-limited scanners, slower protocols relative to the sampling can bias K1 estimates due to poor count statistics per frame. However, our results can be applied for acquisition with any kind of PET system with high count rate capabilities. Furthermore, variations in other methodological factors such as FCH dose, scatter correction, prompt gamma correction, image reconstruction and post-filtering, patient motion, and tracer kinetic modeling could also affect the results.

## Conclusions

A simple two-phase framing of dynamic FCH PET images where the blood phase has frame durations of 5 s and the tissue phase length of 30 s optimally samples TAC for modern PET systems. This time sampling protocol could be used for an optimal FCH uptake quantification in prostate cancer assessment.

## Abbreviations

AIC: Akaike information criterion
FCH: ^18^F-Choline
IDIF: Imaging-derived input function
PC: Prostate cancer
PET/CT: Positron Emission Tomography/Computed Tomography
SUV: Standardized uptake value
TAC: Time-activity curves
VOI: Volume of interest
